# Hesitancy toward Childhood and Influenza Vaccines: Experiences from Highly Educated Jordanian Parents

**DOI:** 10.3390/vaccines12080945

**Published:** 2024-08-22

**Authors:** Montaha Al-Iede, Mohammad Aljahalin, Eva Fashho, Sami Sweis, Rahaf Mesmeh, Loai Bani Hamad, Leen Abuzaid, Jana Al Sa’ed, Yasmeen Elbetar, Aya Yaseen Mahmood Alabdali, Shahed Al-Nawaiseh, Abdallah Al-Ani

**Affiliations:** 1 Division of Pediatric Pulmonology and Sleep Medicine, Department of Pediatrics, Jordan University Hospital, Amman 11942, Jordan; 2 The School of Medicine, The University of Jordan, Amman 11942, Jordan; m.jahalin02@gmail.com (M.A.); evarfashho@gmail.com (E.F.); samisweis10@gmail.com (S.S.); r.mesmeh4@icloud.com (R.M.); yasmeenalbitar.02@gmail.com (Y.E.); 3 The School of Medicine, Al-Balqa’ Applied University, Salt 19117, Jordan; lbanyhamad@gmail.com; 4 The School of Medicine, Yarmouk University, Irbid 21163, Jordan; leen.abuzaid12@gmail.com (L.A.); saed.jana02@gmail.com (J.A.S.); 5 Faculty of Pharmacy, The University of Mashreq, Baghdad 10023, Iraq; ayaalabdaly@hotmail.com; 6 The School of Medicine, Mutah University, Salt 61710, Jordan; shahednawaiseh5@gmail.com; 7 Office of Scientific Affairs and Research, King Hussein Cancer Center, Amman 11943, Jordan; abdallahalany@gmail.com

**Keywords:** hesitancy, influenza, Jordan, PACV, parents

## Abstract

We aimed to examine vaccine hesitancy and knowledge towards influenza vaccines among Jordanian parents. Data were collected via an online questionnaire distributed between October 2023 and March 2024. They included sections on demographics, parental attitudes towards childhood vaccines (PACVs), and knowledge and practices towards influenza vaccines. Associations were examined using the chi-squared test. A binary logistic regression model was utilized to determine predictors of vaccine usage. A total of 3208 participants were included, of which 9.3% were vaccine hesitant per the PACV categorization. Fathers were more likely to be vaccine hesitant (OR: 1.40; 95CI: 1.07–1.85). Similarly, divorced parents (OR: 1.80; 95CI: 1.05–3.12) were significantly more vaccine hesitant compared to their married counterparts. Conversely, higher monthly income (OR: 0.66; 95CI: 0.48–0.92), working in healthcare settings (OR: 0.71; 95CI: 0.51–0.98), and adherence to national vaccination policies (OR: 0.07; 95CI: 0.04–0.13) were significantly associated with a lower likelihood of vaccine hesitancy. Multivariate analysis shows that a healthcare-related occupation (OR: 0.62; 95CI: 0.44–0.87), semi-compliance (OR: 0.37; 95CI: 0.22–0.64), full compliance (OR: 0.08; 95CI: 0.05–0.13) with national vaccine guidelines, and knowledge scores of influenza and vaccines (OR: 0.79; 95CI: 0.75–0.84) were the only independent factors influencing vaccine hesitancy. Finally, non-hesitant participants were significantly more likely to give the influenza vaccine to their children at the present or future time (OR: 2.07; 95CI: 1.53–2.80). Our findings highlight the complexity of vaccine hesitancy and underscore the importance of tailored interventions. Cultural, socioeconomic, and individual factors play significant roles in shaping attitudes toward vaccination. An understanding of the aforementioned among Jordanian parents provides insights for public health initiatives. Compliance with national vaccination guidelines and addressing concerns about vaccine safety are essential for improving childhood vaccination rates in Jordan.

## 1. Introduction

Seasonal influenza is an acute and highly contagious viral respiratory infection that varies in severity and can cause significant morbidity and mortality [1]. The influenza virus’s ability to antigenically switch allows it to spread rapidly from person to person, notably affecting children, who are at the highest risk of catching the virus and developing a severe illness [2,3,4]. Annually, 5–10% of the global adult population experiences an influenza attack, whereas this percentage increases to 20–30% among children, as stated by the World Health Organization (WHO) [5].

Seasonal vaccination programs are one of the most effective tools used to protect individuals and prevent transmission of the influenza virus [6]. The American Centers for Disease Control and Prevention (CDC) recommends annual seasonal influenza vaccinations for anyone above the age of 6 months while prioritizing vaccination for high-risk groups, such as children and immunocompromised patients [7,8]. Despite this, vaccine hesitancy poses a significant global health concern, presenting an obstacle to childhood vaccination efforts. Vaccine hesitancy is defined as the delay in acceptance or refusal to accept vaccination despite readily available vaccination services [9]. This hesitancy may be attributed to several factors such as socioeconomic factors, concerns about vaccination effectiveness and safety, healthcare worker recommendations, and a lack of knowledge and information about the vaccine [1,10].

The Hashemite Kingdom of Jordan is an upper-middle-income country characterized by a population of 10.8 million and a gross domestic product of USD 42.3 billion [11]. The Jordanian population is young, with one-third being under the age of 15 years. Furthermore, the majority of inhabitants reside in the capital city of Amman. In Jordan, the influenza vaccine is not a part of the National Vaccination Program and is, therefore, not mandatory [7,12]. Previous studies have recorded the influenza vaccination rates in Jordan to be between 9.9% and 27.5%; moreover, 63% of the population believed that the influenza vaccine is an important preventive measure for influenza epidemics [1,7]. Interestingly, a 2019 study showed that the majority of older Jordanian adults demonstrate negative attitudes towards the influenza vaccine [13]. Moreover, Zein et al. claim that lobbying by anti-vaccine groups is halting vaccine rollout across Jordan, Palestine, and Syria [14].

While there are numerous studies tackling the knowledge of the Jordanian population regarding the influenza vaccine, studies focusing on parental hesitancy towards giving their children the influenza vaccine are lacking. Therefore, this study aims to better understand the factors influencing parents’ decision to vaccinate their children by looking into influenza vaccine hesitancy in Jordanian parents as well as identify possible barriers that limit parents from vaccinating their children.

## 2. Materials and Methods

### 2.1. Study Design and Population

This was a questionnaire-based cross-sectional study aiming to determine parental hesitancy towards childhood vaccinations, particularly focusing on the influenza vaccine. Data were collected from the participants between October 2023 and March 2024 in Jordan. A Google Form questionnaire was distributed across pediatric clinics across various Jordanian hospitals, including Jordan University Hospital, King Abdullah University Hospital, Aydon Health Centre, Ramtha Hospital, and Al Karak Public Hospital. Parents were handed QR codes which directed them towards the questionnaire should they accept to participate. Furthermore, to ensure an adequate number of participants, a Google Form link was disseminated through multiple social media platforms, such as WhatsApp and Facebook. The data were obtained from parents who lived in Jordan and had at least one child under the age of 18. It should be noted that the source of data collection did not affect this study’s analysis.

### 2.2. Sample Size

The sample size was measured using the following equation: *N* = (Z^2^ × *p* (1 − *p*))/E^2^, where Z = 1.96, *p* = population proportion = 0.5, and E = margin of error = 0.05. Per the aforementioned equation, a minimum of 385 participants was required to conduct statistical analyses of appropriate power. In an effort to maximize the generalizability of the findings, the final sample included a total of 3546 participants.

### 2.3. Questionnaire Design

An online, self-administered, structured Arabic questionnaire was utilized, adapted from a previously validated instrument. The questionnaire underwent validation, including translation from English to Arabic and subsequent back-translation to English [15].

This online questionnaire investigated parents’ attitudes towards childhood vaccination, with a specific focus on the influenza vaccine, and was structured into 3 sections. The first section of this questionnaire consists of 17 items concerning parental and child sociodemographic characteristics. For parents, it explored factors such as age, marital status, family income, and educational level, while for children, it inquired about age, gender, number of children, and vaccination status. The second section delved into parental attitudes towards childhood vaccines (PACVs), comprising 15 items distributed across behavior (2 items), safety and efficacy (4 items), general attitude (4 items), and trust (5 items) domains. Items were scored by assigning a score of 2 for hesitant responses, 1 for unsure responses, and 0 for non-hesitant responses. The sum of all items ranges from 0 to 100 and is categorized into hesitant (score ≥ 50) and non-hesitant (score < 50) [16].

The third section consisted of 10 knowledge items and 5 practice items on influenza and its vaccine. All items in the third section are answered on a 3-response scale (i.e., yes, no, I don’t know). Correct responses were given a score of 1, while incorrect or unsure responses were given a score of 0. This latter section of the questionnaire was adopted from the literature and modified to fit our target audience or avoid redundant questions [17]. In total, the questionnaire comprised 47 items, which took approximately 7–10 min to complete.

### 2.4. Ethical Approval

The study protocol was approved by the Institutional Review Board (IRB) at Jordan University Hospital and the University of Jordan (Ref. No. 2023\28299). Confidentiality was maintained for all the gathered data. Participation in this study was voluntary. After a thorough description of this study’s objectives, all participants were asked to provide their informed consent at the beginning of the questionnaire.

### 2.5. Statistical Analysis

All data management and analyses were conducted on SPSS version 23.0. Descriptive statistics were utilized to showcase the data. Associations between categorical variables were explored using the chi-squared test. Any and all 2 × 2 associations were supplemented with odds ratios (ORs) when applicable. A binary logistic regression model was utilized to assess predictors of vaccine hesitancy. A *p*-value of less than 0.05 was considered statistically significant.

## 3. Results

A total of 3208 participants were included in this study. The greater majority of included parents were mothers (79.4%). Most participants comprised the >40 years age group (45.9%), were married (93.5%), and had a university education or higher (72.1%). While 52.8% had active occupations, only 19.6% worked in healthcare-related jobs. Furthermore, most participants earned between JOD 500 and 1000 per month (41.6%) (Table 1). Finally, only 1.9% of included parents were not compliant with the national recommendations for vaccines; nonetheless, only 15.5% gave their children the COVID-19 vaccine.

Per the assigned threshold for PACV scores, only 9.3% of the included sample were categorized as vaccine “hesitant”. Participants’ responses to the individual items of the PACV questionnaire are presented in Table 2. Notable responses included the following: 9.7% of participants would refuse to give vaccines for reasons other than allergy or illness, 0% of participants agreed that developing immunity by sickness is an alternative to vaccination, 0% concurred that fewer vaccines per unit time are not optimal, 58.3% of participants had concerns over the serious side effects of vaccination, and 47.6% had concerns over the vaccines’ safety in pediatrics. Interestingly, 18.8% of participants had low trust in their pediatrician.

**Figure 1:**
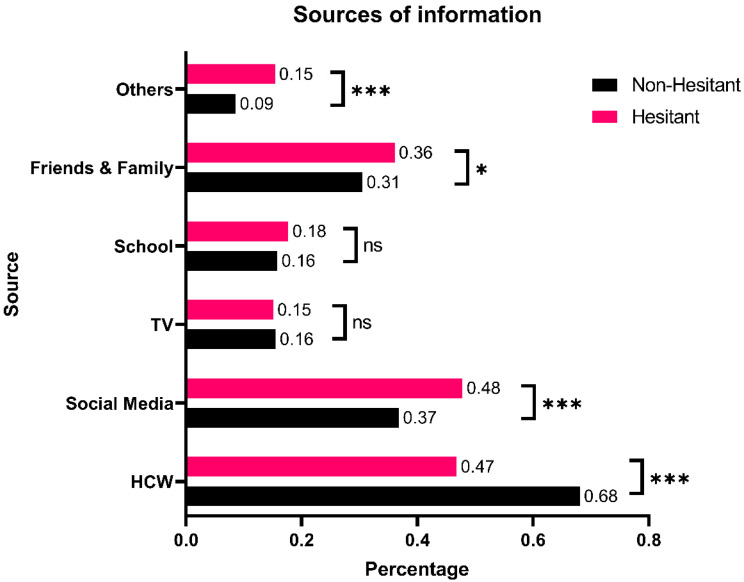
Sources of information about the influenza vaccine and their associations with vaccine hesitancy. * *p* < 0.05, *** *p* < 0.001, ns: not significant.

The following were the sources of information for vaccine-related information in descending order: healthcare workers (66.1%), social media (37.8%), friends and family (31.0%), school (16.0%), and TV (15.5%). Figure 1 demonstrates that non-hesitant participants were significantly more likely to base their vaccine-related information on healthcare worker recommendations (OR: 2.43; 95CI: 1.91–3.08). On the other hand, vaccine-hesitant participants were significantly more likely to extract their vaccine-related information from social media (OR: 1.57; 95CI: 1.24–1.99) and/or friends and family (OR: 1.29; 95CI: 1.01–1.65).

Our univariate analysis demonstrated a number of factors associated with vaccine hesitancy. Compared to mothers, fathers were significantly more likely to be vaccine hesitant (OR: 1.41; 95CI: 1.07–1.85). In reference to married participants, divorced parents were significantly more likely to be vaccine hesitant (OR: 1.81; 95CI: 1.05–3.12). On the other hand, higher levels of income were protective against vaccine hesitancy (*p* = 0.017). Workers in healthcare were significantly less likely to be vaccine hesitant (OR: 0.71; 95CI: 0.51–0.98). Similarly, participants fully or semi-compliant with the national vaccine guidelines for children were significantly less likely to be associated with vaccine hesitancy ([OR: 0.74; 95CI: 0.04–0.13] and [OR: 0.35; 95CI: 0.20–0.60], respectively).

Table 3 demonstrates the knowledge of influenza and its vaccine among included participants. The majority of participants had fairly high knowledge of influenza disease as the majority recognized its serious complications (69.2%), highly contagious nature (94.8%), and transmission route (95.0%). On the other hand, knowledge of the influenza vaccine was rather fair. Only 47.3% regarded the vaccine as effective, 45.0% as safe, and 38.2% believed that influenza vaccines are necessary for children. Interestingly, there was a lack of consensus on whether the vaccine could cause influenza. Finally, 60.8% of participants believed that antibiotics cannot treat viral infections. Mean knowledge scores for the entire cohort were 6.0 (4.0–7.0) points out of 10. Mean knowledge scores were significantly higher for non-hesitant participants (*p* < 0.001).

Multivariate analysis shows that a healthcare-related occupation (OR: 0.62; 95CI: 0.44–0.87), semi-compliance (OR: 0.37; 95CI: 0.22–0.64), full compliance (OR: 0.08; 95CI: 0.05–0.13) with national vaccine guidelines, and higher knowledge scores of influenza and its vaccine (OR: 0.79; 95CI: 0.75–0.84) were the only factors influencing vaccine hesitancy (Table 4). Sensitivity analysis, which included only parents with college degrees, demonstrated that fathers were more likely to be vaccine hesitant (OR: 1.47; 95CI: 1.002–2.17), while having an active occupation (OR: 0.71; 95CI: 0.50–0.99), full compliance with national vaccine guidelines (OR: 0.14; 95CI: 0.06–0.29), and higher knowledge scores of influenza and its vaccine (OR: 0.81; 95CI: 0.75–0.87) were negative predictors of vaccine hesitancy.

When asked about their vaccine practices, 47.7% of parents claim that they had taken the influenza vaccine, 30.5% had provided it for their child in the past, and only 29.9% were willing to give the influenza vaccine to their children at the present or at a future time. Interestingly, the earlier two practices were not significantly associated with vaccine hesitancy (*p* = 0.865 and 0.177, respectively). However, non-hesitant participants were significantly more likely to give the influenza vaccine to their children at the present or at a future time (OR: 2.07; 95CI: 1.53–2.80).

## 4. Discussion

We studied vaccine hesitancy among the Jordanian public using the PACV questionnaire. Our paper’s notable findings include that 9.3% of included participants were considered vaccine hesitant. Such hesitancy was more common when the respondent was a mother. Other factors associated with hesitancy included marital status, income level, healthcare occupation, and previous compliance with national vaccine guidelines. Only the latter two factors remained significant in the multivariate analysis. Furthermore, vaccine hesitancy was associated with the intention to give influenza vaccines in the future. Finally, healthcare workers were the most prevalent source of information on vaccines, and they were significantly associated with less vaccine hesitancy.

Our vaccine hesitancy rate could be considered the lowest among the present literature using the PACV at 9.3%. Rates from similar studies range from 9.8% in Peru to 34.7% in Italy [18,19]. The discordance between rates among different countries is often attributed to differences in study participants and settings. This explanation could be partially true, as the PACV tool itself presents a threshold that is yet to demonstrate its accuracy in predicting actual vaccine-related behaviors. Thus, errors of estimation cannot be ruled out. Another factor that may explain the low rate of hesitancy in our cohort is related to its composition, as most participants had graduate degrees or higher.

Among our participants, concerns mainly revolved around the vaccine’s serious adverse effects and its efficacy in preventing disease. Such concerns are vital in shaping health behaviors, as the previous literature has shown that observing adverse events, whether personally or through other mediums, is associated with a lower likelihood of opting in for vaccination [20]. They may also encourage affected individuals to seek out any sources that might support vaccine-hesitant viewpoints or join chambers of individuals with similar experiences [21]. Within the literature, a higher perception of disease risk is directly correlated with vaccination rate. This might not be the case in our population, as other factors also play a role in mediating adopting preventive behaviors [22]. According to the health belief model, these include perceived severity, benefits, susceptibility, and barriers, among others [23]. Nonetheless, participants with higher knowledge of influenza and its vaccine were more likely to be non-hesitant when it comes to vaccine uptake.

Interestingly, at the level of factors affecting vaccine hesitancy, there is no clear consensus among the published literature. For example, Truong et al. found that unemployment was associated with vaccine hesitancy on both unadjusted and adjusted models [24]. Moreover, the NAVIDAD Italian study found a higher prevalence of vaccine hesitancy among highly educated mothers living in high income brackets [25]. The opposite of the aforementioned findings was observed in our study. This might suggest that while the PACV questionnaire is a structurally and psychometrically solid tool to gauge a general understanding of vaccine hesitancy, it might not capture the entire spectrum of such social phenomenon, as it lacks the cultural modifications to do so. Another explanation for the observed differences may simply lie within the differences in the sampling techniques implemented within all referenced studies.

Similar to the findings of Alsuwaidi et al. in their exploration of vaccine hesitancy in the United Arab Emirates [15], our study found a significant link between “divorced” marital status and higher likelihood of vaccine hesitancy. Such a link serves as a proxy for the impact of family structure on the health of children. Jane Anderson reports that divorce affects children by impacting their economic security, emotional security, psychological and social maturation, outlook on sexual behavior, religious faith, cognitive and academic stimulation, physical health, and mental health [26]. Jordan ranks third in the highest country with divorce rates in the Arab world at 37.2% [27]. Thus, social workers and healthcare personnel should exercise extra care and consideration when dealing with pediatrics within separated families.

In our study, mothers were significantly less vaccine hesitant than fathers. Such observation is well documented in the literature examining health behaviors, as studies using the health belief model demonstrated that females are more likely to adopt, support, and comply with preventive measures in response to or in anticipation of health hazards [22,28]. This finding has serious legal and ethical implications. Within Jordanian society, it is the legal duty of males to make health decisions within the nuclear family (e.g., approving the COVID-19 vaccine for children). A higher vaccine hesitancy rate among males may lead to reduced vaccine rollout despite the growing awareness of their importance among mothers. Parental approval was so deep of an issue that the Jordanian Ministry of Health, in 2023, planned for a nationwide vaccine roll against measles and rubella for pediatrics, irrespective of parental consent. While the intentions of the Ministry might not be malicious, they serve as violations of autonomy. Nonetheless, ElSayed et al. found and described a range of conflicting results on the association of biological sex with vaccine hesitancy in the Arab region [29].

A striking finding within our study is the dissonance between measured vaccine hesitancy and actual vaccine behavior. There was no association between hesitancy and previous vaccination status of either parents or children. However, non-hesitancy was associated with more favorable views on future influenza vaccination. The earlier cognitive dissonance has been implicated in vaccine hesitancy [30]. However, the Harmon–Jones theory of the Action-Based Model of Dissonance perfectly encapsulates this phenomenon [31]. Due to the spread of misinformation, an individual could hold two conflicting ideas, which inherently involve a psychological construct termed “action tendency”. This state is uncomfortable and interferes with the ability to take effective action; thus, it produces a dissonance between “good” belief and “bad” action (i.e., believing in the vaccine but not giving it to one’s children). However, this negative affective state motivates the organism to engage in behaviors that correct the resultant problem by selecting certain action tendencies until all interferences with action are eliminated, the result of which is exemplified in the increased tendency of included parents to vaccinate their children in the future.

Away from psychology and back to public health basics, vaccine hesitancy was associated with social media and close social circles (i.e., friends and family), while non-hesitancy was associated with taking information from healthcare workers. These findings funnel heavily into the possible recommendations to tackle vaccine hesitancy by policymakers and concerned authorities. Firstly, policymakers and concerned bodies should aim to expand the scope, intensity, and frequency of awareness campaigns among the general public through all possible mediums with a special focus on trustworthy organizations [32]. Additionally, concerned authorities should strive to control and strike false information heavily advertised through social media and the internet [28]. While posing many ethical, legal, and practical challenges, vaccine mandates have proven their effectiveness in small- to medium-scale nations [33]. Furthermore, resources should be allocated to provide parents with incentives to vaccinate their children.

Our findings should be interpreted within the context of the following limitations. Firstly, this was a cross-sectional study that is associated with inherent limitations, such as “snap shot” sampling and the inability to assess causal and/or temporal relationships. Secondly, while this study employed a validated questionnaire, the close-end nature of the questionnaire and its lack of a validated threshold may have led to the loss of response range and errors in estimation, respectively. Thirdly, certain biases were not accounted for, including recall bias and/or social desirability bias. Fourthly, an online-only data collection may have attracted only a subset of the Jordanian population who have access to the internet, mainly those who are educated, young, and have a higher income. Fifthly, the convenient sampling of participants may have led to a sample, despite its relatively large size, which may not be representative of the Jordanian public. Finally, this study could not assess actual vaccine-related behaviors prior to or after questionnaire dissemination.

## 5. Conclusions

Using the PACV questionnaire, our study reveals a relatively low vaccine hesitancy rate of 9.3% among Jordanians, particularly among those who are highly educated. This rate is significantly lower than comparable research conducted in other countries, potentially due to the participants’ higher educational levels. A healthcare occupation and compliance with national vaccine guidelines were significant factors influencing vaccine hesitancy. These findings underscore the important role that healthcare workers play by serving as a source of information.

Our study also highlights the complexity of vaccine reluctance and the impact of psychological factors, such as cognitive dissonance. Reliance on healthcare professionals was linked to non-reluctance, while social media and intimate social circles were identified as sources of hesitancy. These findings indicate that vaccine hesitancy requires a variety of strategies, such as raising public knowledge through reliable sources, controlling false information, and taking culturally appropriate interventions into account.

## Figures and Tables

**Table 1:** Characteristics of included participants stratified by vaccine hesitancy status.

		Non-Hesitant	Hesitant		
Variable	Category	*N* (%)	*N* (%)	*p*-Value	Odds Ratio (95% CI)
Parent	Father	583 (20.0%)	78 (26.1%)	0.014	1.408 (1.071–1.851)
	Mother	2326 (80.0%)	221 (73.9%)		
Age group	20–30	550 (18.9%)	69 (23.1%)	0.193	REF
	30–40	1014 (34.9%)	103 (34.4%)		0.810 (0.587–1.117)
	>40	1345 (46.2%)	127 (42.5%)		0.753 (0.552–1.026)
Marital status	Married	2732 (93.9%)	269 (90.0%)	0.029	REF
	Divorced	90 (3.1%)	16 (5.4%)		1.806 (1.046–3.118)
	Widow	87 (3.0%)	14 (4.7%)		1.634 (0.917–2.913)
Educational level	None	35 (1.2%)	5 (1.7%)	0.013	NA
	Primary	41 (1.4%)	10 (3.3%)		NA
	Foundational	90 (3.1%)	16 (5.4%)		NA
	High school	629 (21.6%)	69 (23.1%)		NA
	University	2114 (72.7%)	199 (66.6%)		NA
Occupation	Does not work	1363 (46.9%)	151 (50.5%)	0.229	0.864 (0.681–1.096)
	Works	1546 (53.1%)	148 (49.5%)		
Income level	<500	980 (33.7%)	125 (41.8%)	0.017	REF
	500–1000	1222 (42.0%)	114 (38.1%)		0.731 (0.560–0.956)
	>1000	707 (24.3%)	60 (20.1%)		0.665 (0.482–0.919)
Parent works in healthcare	No	2304 (79.8%)	251 (84.8%)	0.039	0.707 (0.508–0.984)
	Yes	584 (20.2%)	45 (15.2%)		
Number of children	1	470 (16.2%)	48 (16.1%)	NA	NA
	2–4	1727 (59.4%)	162 (54.2%)		NA
	5–7	629 (21.6%)	72 (24.1%)		NA
	>8	83 (2.9%)	17 (5.7%)		NA
Does the child have a chronic disease	No	2685 (92.3%)	269 (90.0%)	0.155	1.337 (0.895–1.997)
	Yes	224 (7.7%)	30 (10.0%)		
Compliance with national vaccine	No	32 (1.1%)	28 (9.4%)	<0.001	REF
	Yes	2523 (86.7%)	164 (54.8%)		0.074 (0.044–0.126)
	Some	354 (12.2%)	107 (35.8%)		0.345 (0.199–0.600)
Compliance with COVID-19 vaccine	No	2464 (84.7%)	247 (82.6%)	0.341	1.166 (0.850–1.598)
	Yes	445 (15.3%)	52 (17.4%)		
PACV score		21.1 ± 12.5	56.1 ± 6.5	<0.001	NA

**Table 2:** Participants’ responses to the PACV questionnaire.

Question	Responses	*N*	%
Have you ever delayed having your child get a shot for reasons other than illness or allergy	Yes	2597	84.8%
	No	467	15.2%
Have you ever decided not to have your child get a shot for reasons other than illness or allergy	Yes	2816	90.3%
	No	304	9.7%
If you had another infant today, would you want him/her to get all the recommended shots	Yes	2707	84.4%
	IDK	246	7.7%
	No	255	7.9%
How sure are you that following the recommended shot schedule is a good idea for your child	8–10	2300	71.7%
	6–7	360	11.2%
	0–5	548	17.1%
Children get more shots than are good for them	Disagree	1496	46.6%
	Not Sure	1073	33.4%
	Agree	639	19.9%
I believe that many of the illnesses shots prevent are severe	Agree	2482	77.4%
	Not Sure	530	16.5%
	Disagree	196	6.1%
It is better for my child to develop immunity by getting sick than to get a shot	Disagree	3208	100.0%
	Not Sure	0	0.0%
	Agree	0	0.0%
It is better for children to get fewer vaccines at the same time	Disagree	3208	100.0%
	Not Sure	0	0.0%
	Agree	0	0.0%
How concerned are you that your child might have a serious side effect from a shot	Not Concerned	876	27.3%
	Not Sure	461	14.4%
	Concerned	1871	58.3%
How concerned are you that any one of the childhood shots might not be safe	Not Concerned	1032	32.2%
	Not Sure	649	20.2%
	Concerned	1527	47.6%
How concerned are you that a shot might not prevent the disease	Not Concerned	1054	32.9%
	Not Sure	1027	32.0%
	Concerned	1127	35.1%
Overall, how hesitant about childhood shots would you consider yourself to be	Not Hesitant	2355	73.4%
	Not Sure	317	9.9%
	Hesitant	536	16.7%
I trust the information I receive about shots	Agree	2299	71.7%
	Not Sure	649	20.2%
	Disagree	260	8.1%
I am able to openly discuss my concerns about shots with my child’s doctor	Agree	2528	78.8%
	Not Sure	513	16.0%
	Disagree	167	5.2%
All things considered, how much do you trust your child’s doctor	8–10	1993	62.1%
	6–7	613	19.1%
	0–5	602	18.8%

**Table 3:** Participants’ knowledge responses of influenza and influenza vaccine.

Question	No	Yes	I Don’t Know
Do you think influenza can lead to serious complications (e.g., Hospitalization or death)	608 (19.0%)	2221 (69.2%)	379 (11.8%)
Do you think influenza is a highly contagious disease?	98 (3.1%)	3042 (94.8%)	68 (2.1%)
Do you know influenza is transmitted primarily by coughing and sneezing?	81 (2.5%)	3048 (95.0%)	79 (2.5%)
Do you think the influenza vaccine is effective in preventing the flu?	969 (30.2%)	1516 (47.3%)	723 (22.5%)
Do you think the influenza vaccine is safe?	671 (20.9%)	1443 (45.0%)	1094 (34.1%)
Do you think the influenza vaccine is necessary for children?	1184 (36.9%)	1227 (38.2%)	797 (24.8%)
Do you know the influenza vaccine is recommended for all children 6 months of age or older?	1000 (31.2%)	1223 (38.1%)	985 (30.7%)
The influenza vaccine will cause influenza	902 (28.1%)	1059 (33.0%)	1247 (38.9%)
The influenza vaccine should be given every year	913 (28.5%)	1243 (38.7%)	1052 (32.8%)
Antibiotics cannot treat a viral infection	618 (19.3%)	1950 (60.8%)	640 (20.0%)

**Table 4:** Multivariate analysis of predictors of vaccine hesitancy.

		*p*-Value	OR	L95% CI	U95% CI
Parent	Parent (Father)	0.203	1.214	0.901	1.636
Age of parent	20–30	0.963	REF		
	30–40	0.964	1.008	0.721	1.408
	>40	0.809	1.043	0.740	1.470
Marital status	Married	0.478			
	Divorced	0.231	1.416	0.801	2.502
	Widowed	0.781	1.091	0.591	2.014
Educational level	None	0.915	REF		
	Primary	0.812	1.166	0.330	4.122
	Foundational	0.833	1.132	0.357	3.587
	High school	0.819	0.885	0.311	2.517
	University	0.959	0.973	0.345	2.742
Occupation	Occupation (Working)	0.413	0.892	0.678	1.173
Income level	<500	0.243	REF		
	500–1000	0.097	0.773	0.571	1.047
	>1000	0.263	0.814	0.569	1.166
Working in healthcare	Healthcare (Works)	0.006	0.623	0.444	0.874
Number of children	1	0.387	REF		
	2–4	0.483	1.132	0.801	1.598
	5–7	0.247	1.273	0.846	1.916
	>8	0.110	1.711	0.885	3.310
A child with a chronic disease	Chronic Disease (True)	0.697	1.086	0.717	1.645
Given vaccines per national program	No	0.000	REF		
	Yes	0.000	0.078	0.046	0.131
	Some	0.000	0.375	0.218	0.643
Given COVID-19 vaccine	COVID-19 Vaccine (True)	0.468	1.129	0.814	1.565
Knowledge of influenza and vaccines		0.000	0.795	0.751	0.841

## Data Availability

Data will be provided at a reasonable request from the corresponding author.
